# Impact of maternal SARS-CoV-2 booster vaccination on blood and breastmilk antibodies

**DOI:** 10.1371/journal.pone.0287103

**Published:** 2023-06-13

**Authors:** Anne-Marie Rick, Anthony Lentscher, Lingqing Xu, Maris S. Wilkins, Amro Nasser, Dylan J. Tuttle, Christina Megli, Ernesto T. A. Marques, Anita K. McElroy, John V. Williams, Judith M. Martin

**Affiliations:** 1 Department of Pediatrics, University of Pittsburgh School of Medicine, Pittsburgh, Pennsylvania, United States of America; 2 University of Pittsburgh School of Medicine, Pittsburgh, Pennsylvania, United States of America; 3 Center for Vaccine Research, University of Pittsburgh School of Medicine, Pittsburgh, Pennsylvania, United States of America; 4 Department of Infectious Diseases and Microbiology, University of Pittsburgh School of Public Health, Pittsburgh, Pennsylvania, United States of America; 5 Department of Obstetrics, Gynecology and Reproductive Sciences, University of Pittsburgh School of Medicine, Pittsburgh, Pennsylvania, United States of America; 6 Magee Womens Research Institute, Pittsburgh, Pennsylvania, United States of America; Central University of Tamil Nadu, INDIA

## Abstract

Maternal COVID-19 vaccination could protect infants who are ineligible for vaccine through antibody transfer during pregnancy and lactation. We measured the quantity and durability of SARS-CoV-2 antibodies in human milk and infant blood before and after maternal booster vaccination. Prospective cohort of lactating women immunized with primary and booster COVID-19 vaccines during pregnancy or lactation and their infants. Milk and blood samples from October 2021 to April 2022 were included. Anti-nucleoprotein (NP) and anti-receptor binding domain (RBD) IgG and IgA in maternal milk and maternal and infant blood were measured and compared longitudinally after maternal booster vaccine. Forty-five lactating women and their infants provided samples. 58% of women were anti-NP negative and 42% were positive on their first blood sample prior to booster vaccine. Anti-RBD IgG and IgA in milk remained significantly increased through 120–170 days after booster vaccine and did not differ by maternal NP status. Anti-RBD IgG and IgA did not increase in infant blood after maternal booster. Of infants born to women vaccinated in pregnancy, 74% still had positive serum anti-RBD IgG measured on average 5 months after delivery. Infant to maternal IgG ratio was highest for infants exposed to maternal primary vaccine during the second trimester compared to third trimester (0.85 versus 0.29; p<0.001). Maternal COVID-19 primary and booster vaccine resulted in robust and long-lasting transplacental and milk antibodies. These antibodies may provide important protection against SARS-CoV-2 during the first six months of life.

## Introduction

Children <6 months old were excluded from SARS-CoV-2 vaccine trials and are not eligible for vaccine in the United States [1]. However, infants <12 months with COVID-19 are more likely to require hospitalization, respiratory support, intensive care and are at higher risk of death compared to children 1–4 years old [2–5]. Strategies to protect this vulnerable population are necessary.

Transplacental acquisition of IgG following maternal immunization protects infants from tetanus, pertussis and influenza during the first 6 months of life [6–9]. Immunization of women during pregnancy with COVID-19 vaccines results in trans-placental transfer of anti-receptor binding domain (RBD) and neutralizing IgG antibodies [10]. Both of these antibodies are known correlates of protection in adults and could provide protection against infant infections. In fact, maternal immunization with COVID-19 vaccines during pregnancy is associated with a 50% decreased risk of infant hospitalization with COVID-19 [11]. While it is hypothesized that transplacental antibodies against COVID-19 are likely present and protective through approximately six months of life, there is limited data examining the durability of these antibodies following delivery. Furthermore, anti-RBD IgG and IgA antibodies are detectable in human milk from lactating women after SARS-CoV-2 primary vaccination for up to six months [10, 12–15], and virus-neutralizing antibodies are detected in saliva and stool of breastfed infants [15, 16]. However, the ability of milk antibodies to protect infants against COVID-19 is less understood. Furthermore, limited data exists on the impact of maternal booster vaccination on milk and infant blood antibody titers [17].

In this study, we measured the quantity and durability of anti-RBD SARS-CoV-2 antibodies in human milk and maternal and infant blood prior to and following maternal booster vaccine with ancestral strain. We hypothesized that infants born to women vaccinated in pregnancy would still have persistent anti-RBD in their blood and that booster vaccine but substantially increase milk anti-RBD compared to pre-booster samples but would not change infant blood titers. This will provide critical information that can be used to by clinicians to tailor vaccine counselling for pregnant and lactating women who may be motivated by potential benefits to their child.

## Methods

### Study population

This study used data obtained from a prospective cohort of lactating women and their children recruited from April 2021 to September 2021 to provide longitudinal human milk samples up to one year after primary COVID-19 vaccine. For the parent study, eligible women were ≥ 18 years, lactating, and were vaccinated or planning to be vaccinated with any COVID-19 vaccine at time of enrollment. Starting in September 2021, participants were invited to partake in sub-study collecting longitudinal blood samples in addition to milk. For this analysis, we included data from women and their infants who 1) consented to participate in both studies, 2) mother received a complete primary COVID-19 vaccines series, 3) mother obtained a COVID-19 booster vaccine and 4) had at least one blood and milk sample available before and 15–35 days after booster vaccine. The University of Pittsburgh Institutional Review Board approved this study and all participants provided written informed consent (STUDY21010154 and STUDY21080124).

### Data and sample collection

All women were asked to provide up to five fresh or frozen milk samples every 3 months (+/- 1 month) starting before their first COVID-19 vaccine or at delivery if vaccinated while pregnant. For women who received a COVID-19 booster vaccine while lactating and opted to provide blood samples, additional paired milk and blood samples (mother and infant) were collected <30 days before and 14–35 days after receiving a booster. Women then resumed routine milk collection schedule as outlined at enrollment. A convenience sample of mother-infant pairs were requested to provide a third blood sample with a milk sample occurring 60–119 days or 120–170 days after booster vaccine. Detailed milk and blood collection methods are included in S1 File. All paired milk and blood samples as well as any milk samples collected in the interval between blood samples were included in this analysis. In addition, baseline and COVID-19 infection data obtained from self-reported online surveys completed by participants with each milk collection were included.

### Enzyme-linked immunosorbent assay (ELISA) for SARS-CoV-2 nucleoprotein and receptor binding domain

Using indirect ELISA, we assessed serum IgG binding to SARS-COV-2 nucleoprotein (NP) as previously described and detailed in S1 File. [18] The assay was optimized to detect anti-SARS-CoV-2 NP IgG with sensitivity of 93.4% and specificity of 89.3%. [18] SARS-CoV-2 RBD IgG and IgA ELISA were performed as described with modifications defined in Supplemental Material. [19] This assay was optimized to detect anti-SARS-CoV-2 RBD IgG (cut-off titer ≥ 900) with sensitivity of 94.8% and specificity of 97.8% and IgA (cut-off titer ≥ 300) with sensitivity of 88.2% and specificity of 97.3% in blood. The limit of detection on this assay was 100; samples below the limit were set to 99 for statistical analysis. Positive cut-off values for milk IgG (titer ≥ 4; specificity of 93.3%) and secretory IgA (titer ≥ 8: specificity of 96.7%) were determined using 30 pre-pandemic milk samples collected from 2018–2019 obtained from a human milk biobank. The limit of detection for the milk assay was 1.0; samples below this limit were set to 0.9 for statistical analysis.

### Statistical analysis

#### Antibodies in maternal blood and milk by maternal NP status

Using the anti-NP results from the first maternal blood sample, women were divided into two groups: 1) women with no anti-NP antibodies and no reported COVID-19 infection; and 2) women with anti-NP antibodies in their first sample +/- a history of COVID-19 infection. If a woman demonstrated seroconversion to positive anti-NP antibodies between blood samples, reported a new SARS-CoV-2infection, or stopped lactating, all samples obtained at that time or later were excluded. By anti-NP status, we compared the proportion of women who were positive for anti-RBD IgG and IgA in blood and milk at each timepoint using Fisher’s Exact test. We compared milk and blood anti-RBD geometric mean titer (GMT) over time using mixed-effects models with Geisser-Greenhouse correction and Sidak’s multiple comparisons test. First, we assessed models with interaction terms between maternal anti-NP serostatus and time; models with non-significant interaction terms (p>0.05) were removed and the simpler model including anti-NP serostatus and time but no interaction term was used. We used Pearson’s correlation to assess correlations between paired blood and milk titers.

#### Antibodies in infant blood by timing of maternal primary COVID-19 vaccine

To assess differences in infant serum anti-RBD antibody over time, we grouped infants by the timing of their mother’s primary COVID-19 vaccine series, either during pregnancy or lactation. Any samples from infants positive for anti-NP, who had COVID-19, or no longer received breastmilk were excluded. We then compared the proportion of samples positive for IgG or IgA prior to maternal COVID-19 booster vaccine by timing of the mother’s first vaccine using Fisher’s exact test. We then compared blood anti-RBD GMT by timing of the mother’s first vaccine over time using a mixed-effects model with interaction and Geisser-Greenhouse correction and Sidak’s multiple comparisons test.

Using only mother-infant pairs who were both anti-NP negative on their first blood sample, we then assessed the infant to maternal ratio of anti-RBD IgG among infants born to women vaccinated during pregnancy based on trimester of vaccination (2^nd^ trimester defined as 13 to 26 weeks, 3^rd^ as 27 weeks to 41 weeks) using Student t-test. Mother-infant pairs vaccinated in the first trimester were excluded due to small numbers. All statistical analyses were completed on GraphPad Prism 9 or STATA 17 and original deidentified data are available in S2 File.

## Results

### Study population

**Fig 1:**
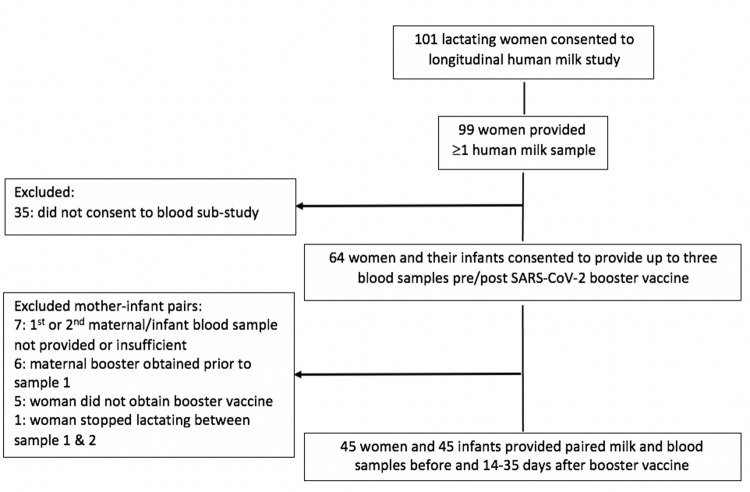
Consort diagram of participants from parent study included in current analysis.

Forty-five women and their children met inclusion criteria for this analysis (Fig 1). Initial blood samples were collected from 10/7/2021 to 1/17/2022 with 96% collected prior to any local SARS-CoV-2 Omicron variant circulation which was first reported 12/12/2021. [20] Women were predominately white, college-educated, and in their mid-30s (Table 1). Fifty-four percent (24/45) of women received primary SARS-CoV-2 vaccine during pregnancy and 95% (43/45) were boosted with the same vaccine type (median time primary to booster: 8.2 months; range 5.8–11.1; SD:1.2). Median time from birth to booster vaccine for women first vaccinated during pregnancy was 5.2 months (range 3.3–7.7; SD: 1.3). All infants born to women vaccinated in pregnancy were ≥ 37 weeks gestational age (Table 1).

**Table 1:** Maternal and infant baseline characteristics by timing of administration of the primary COVID-19 vaccine series to the mother.

Characteristic	Pregnant	Lactation
	N = 24	N = 21
Maternal age, mean (range), y	33.6 (29.2–39.1)	35.6 (29.7–43.2)
Maternal race		
White	24 (100)	20 (95)
Asian	0 (0)	1 (5)
Highest level of education		
Some college	0 (0)	1 (5)
2 or 4 year college	6 (25)	11 (52)
Graduate or professional degree	18 (75)	9 (43)
Employment type		
Healthcare	16 (67)	5 (24)
Non-healthcare	8 (33)	12 (57)
None outside home	0 (0)	4 (19)
Maternal chronic medical conditions		
Asthma	4 (17)	4 (19)
Obesity (BMI > 30)	1 (4)	4 (19)
Autoimmune disorder/cancer	2 (8)	3 (15)
Diabetes	0 (0)	1 (5)
Hypertension or cardiac disease	0 (0)	1 (5)
Medical conditions in last pregnancy		
Gestation hypertension/pre-eclampsia	2 (8)	6 (29)
Gestational diabetes	1 (4)	5 (24)
Clinical COVID-19 infection prior to 1^st^ blood sample		
Mother	0 (0)	1 (5)
Child	0 (0)	0 (0)
Maternal primary COVID-19 vaccine		
BNT162b2 (Pfizer-BioNTech)	10 (42)	8 (38)
mRNA-1273 (Moderna)	14 (58)	11 (52)
Ad26.COV2.S (Janssen/Johnson and Johnson)	0 (0)	2 (100)
Maternal booster SARS-CoV-2 vaccine		
BNT162b2 (Pfizer-BioNTech)	9 (37)	8 (38)
mRNA-1273 (Moderna)	15 (63)	13 (62)
Child sex		
Male	11 (46)	9 (43)
Female	13 (54)	12 (57)
Child gestational age at birth		
< 35 weeks	0 (0)	2 (10)
35–36 weeks	0 (0)	4 (19)
>37 weeks	24 (100)	15 (71)
Child age at 1^st^ blood sample		
< 3 months	2 (8)	0 (0)
≥ 3 to 5 months	17 (71)	1 (5)
≥ 6 to 11 months	5 (21)	7 (33)
≥ 12 to 17 months	0 (0)	8 (38)
≥ 18 months	0 (0)	5 (24)

N (%). BMI: body mass index

### Anti-NP antibody in maternal blood

**Fig 2:**
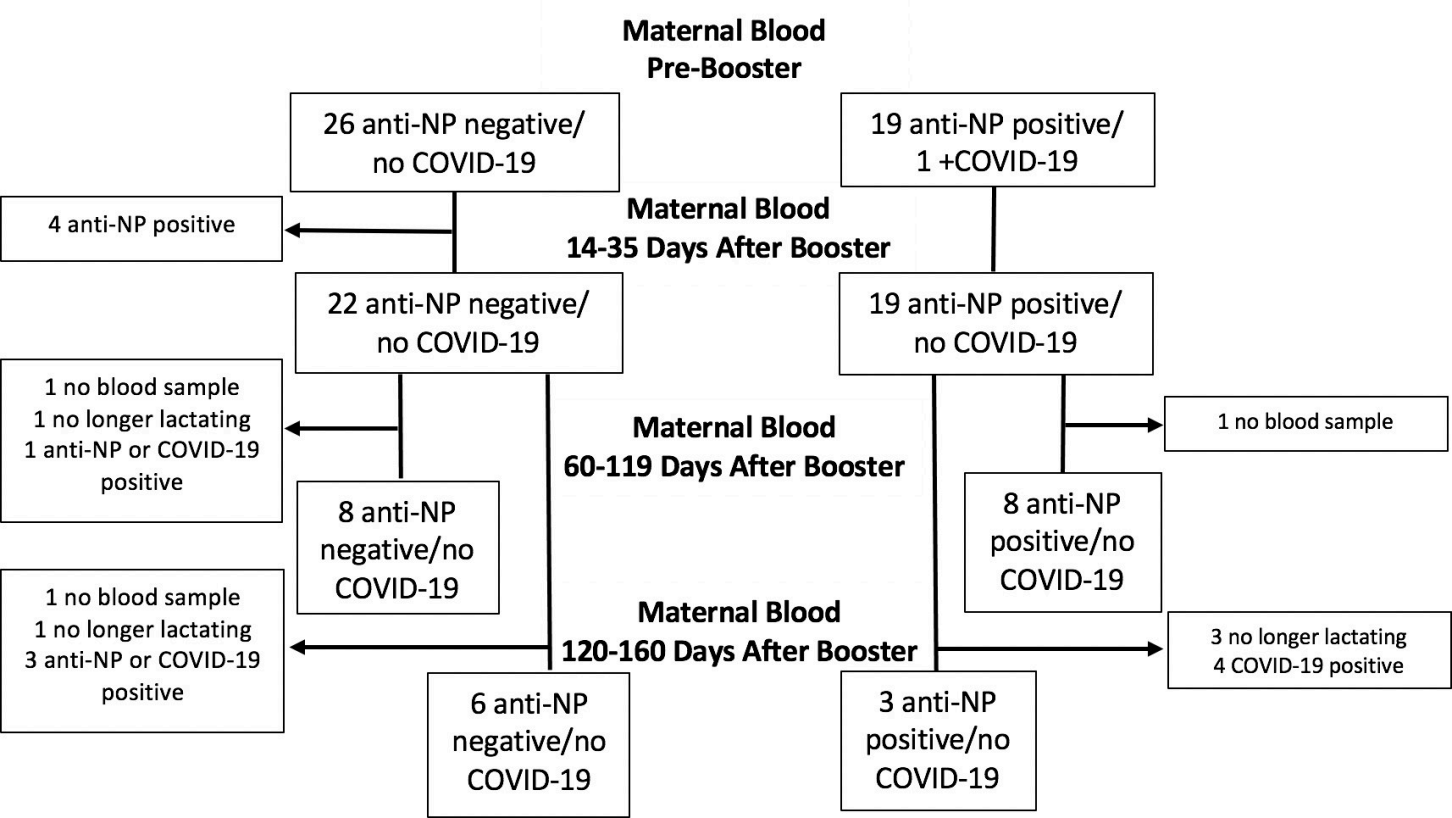
Flow chart of anti-NP results and COVID-19 infections in samples of women included in study (N = 45).

Fifty-eight percent (26/45) of women were negative and 42% (19/45) were positive for anti-NP antibody on their first blood sample obtained prior to booster vaccine (Fig 2). Of those negative, over two-thirds remained negative at subsequent time points. Of those anti-NP positive on the first blood sample, only one woman had known prior clinical infection.

### Anti-RBD antibodies in maternal blood before and after COVID-19 booster vaccine

**Fig 3:**
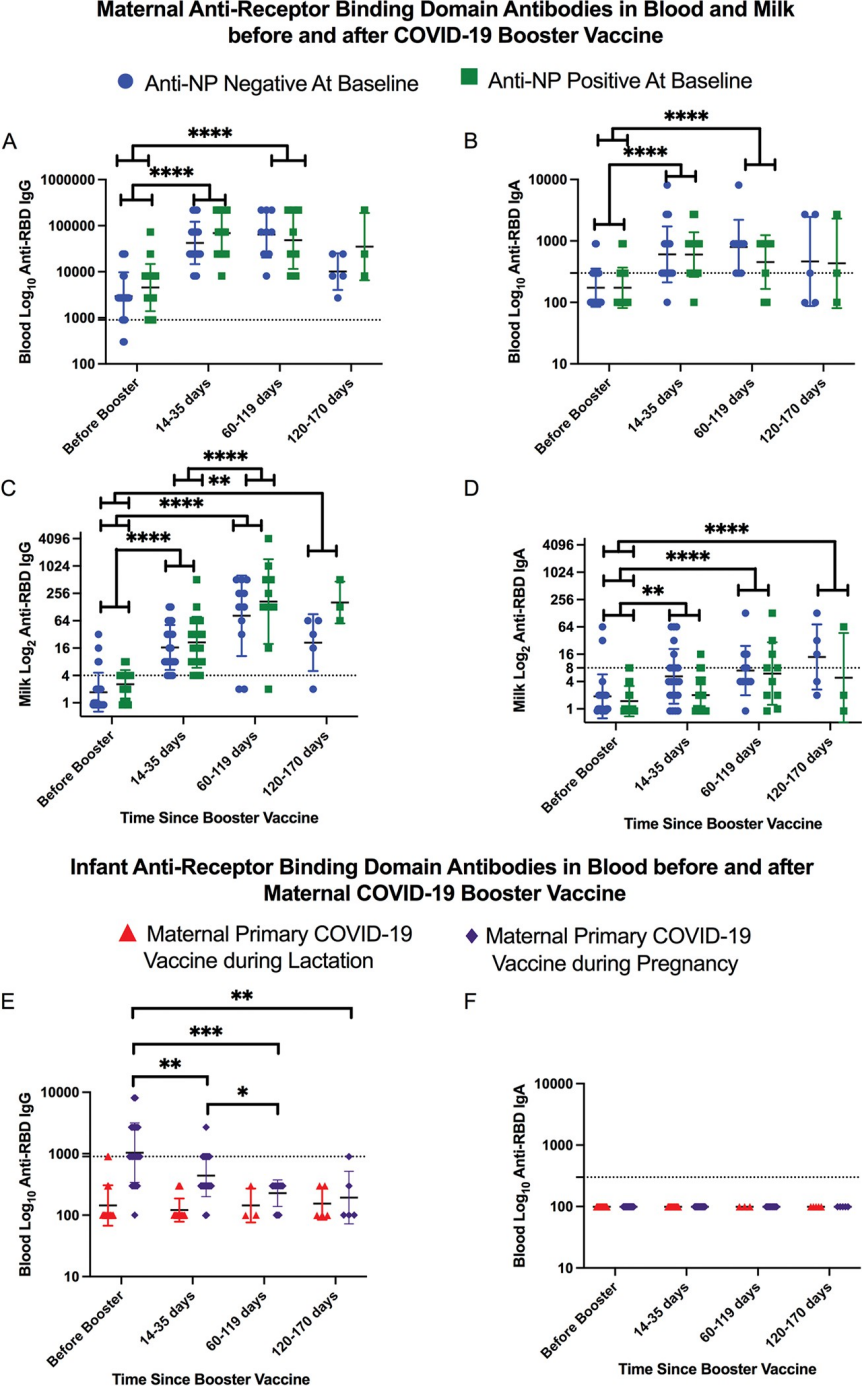
**A-F.** SARS-CoV-2 anti-receptor binding domain (RBD) IgG and IgA geometric mean titer (GMT) in maternal and infant blood and milk before and after maternal COVID-19 booster vaccine (N = 45 pairs). The dotted lines indicate the positive cutoff titer of 900 for IgG and 300 for IgA in blood (A,B, E, F) and 4 for IgG and 8 for IgA in milk (C, D). Mixed effects models with Geisser-Greenhouse correction and Sidak’s multiple comparisons test was used to assess differences over time. Error bars indicate GMT standard deviation. Timepoint comparisons not significant unless otherwise shown; *: p<0.05; **p<0.01; ***p<0.001; **** p<0.0001. Anti-RBD IgG and IgA was significantly increased in maternal blood through 60–119 days after booster vaccine and in milk through 120–170 days after booster vaccine (A-D) regardless of maternal NP status. Anti-RBD IgG GMT in milk continued to increase between 15–35 days and 60–119 days after vaccination (C). Infants of women vaccinated during lactation showed no increase in serum anti-RBD IgG or IgA antibodies after maternal booster vaccine (E- F). Infants of women vaccinated during pregnancy showed a decrease in serum anti-RBD IgG between samples (E).

Ninety-six percent (43/45) of women had positive serum anti-RBD IgG on average eight months after primary series but before booster (Table 2). In contrast, only 40% (18/45) had serum anti-RBD IgA. There was no difference in the proportion of women positive for anti-RBD IgG or IgA or in GMT at any time by maternal serum anti-NP status (Table 2; Fig 3A and 3B). However, regardless of anti-NP status, anti-RBD IgG and IgA GMT in maternal blood significantly increased 2–4 weeks after booster and remained increased through 60–119 days after booster (Fig 3A and 3B). By 120–170 days, there was no difference in GMT compared to pre-booster samples.

**Table 2:** Comparison of the proportion of positive blood and milk samples for anti-RBD IgG and IgA by maternal anti-NP status in blood using Fisher’s exact tests.

	**Blood anti-RBD IgG**				**Milk anti-RBD IgG**			
**Time**	**Anti-NP-**	**Anti-NP+**	**OR (95%CI)**	**p-value** ^**a**^	**Anti-NP-**	**Anti-NP+**	**OR (95%CI)**	**p-value** ^**a**^
Pre-booster	24/26 (92)	19/19 (100)	--^b^	0.50	4/26 (15)	10/19 (53)	0.2 (0.0–0.8)	0.01
Post-booster 14-35d	22/22 (100)	19/19 (100)	--	--	22/22 (100)	19/19 (100)	--	--
Post-booster 60-119d	9/9 (100)	8/8 (100)	--	--	9/11 (82)^c^	9/10 (90)^c^	0.5 (0.0–11.6)	1.00
Post-booster 120-170d	5/5 (100)	3/3 (100)	--	--	4/5 (80)	3/3 (100)	--	1.00
	**Blood anti-RBD IgA**				**Milk anti-RBD IgA**			
**Time**	**Anti-NP-**	**Anti-NP+**	**OR (95%CI)**	**p-value** ^**a**^	**Anti-NP-**	**Anti-NP+**	**OR (95%CI)**	**p-value** ^**a**^
Pre-booster	11/26 (42)	7/19 (37)	1.3 (0.3–5.1)	0.76	3/26 (12)	2/19 (11)	1.1 (0.1–14.6)	1.00
Post-booster 14-35d	21/22 (95)	18/19 (95)	1.2 (0.0–95.9)	1.00	9/22 (41)	4/19 (21)	2.6 (0.5–14.1)	0.20
Post-booster 60-119d	9/9 (100)	6/8 (75)	--	0.21	5/11 (45)^c^	4/10 (40)^c^	1.3 (0.2–9.9)	1.00
Post-booster 120-170d	3/5 (60)	2/3 (67)	0.8 (0.0–27.7)	1.00	3/5 (60)	1/3 (33)	3 (0.1–235.0)	1.00

n/N (%); RBD = Receptor Binding Domain; NP = nucleocapsid; OR: odds ratio; CI: confidence interval; d = days

^a^p-values were calculated using Fisher’s Exact tests.

^b^“--" indicates that a confidence interval or p-value could not be calculated due to zero counts in one or more cells.

^c^Four women (two anti-NP negative; two anti-NP positive) provided unpaired milk samples between their second and third blood sample.

### Anti-RBD antibodies in milk before and after SARS-CoV-2 booster vaccine

Fifteen percent (4/26) of anti-NP negative women and 53% (10/19) of anti-NP positive women had positive milk anti-RBD IgG obtained before booster (Odds ratio (OR) 0.2 (95%Confidence interval (CI) 0.0–0.8; p = 0.011; Table 2). However, after booster, there were no differences in milk anti-RBD IgG or IgA by maternal anti-NP status (Table 2). Using the mixed-effects model, there were no statistical differences in milk IgG and IgA GMT by maternal anti-NP status over time (Fig 3C and 3D). However, there was a non-significant trend toward decreased milk IgG GMT by 120–170 days post-booster among anti-NP negative women (Fig 3C). Among all women, regardless of anti-NP status, milk anti-RBD IgG and IgA GMT significantly increased 14–35 days after booster and remained elevated at all subsequent timepoints relative to the pre-booster sample (Fig 3C and 3D). Milk IgG GMT continued to rise at 60–119 days relative to 14–35 days after booster, with an average 2.6-fold dilution increase (Fig 3C, p<0.0001).

### Correlation of anti-RBD antibodies in maternal blood and milk

**Fig 4:**
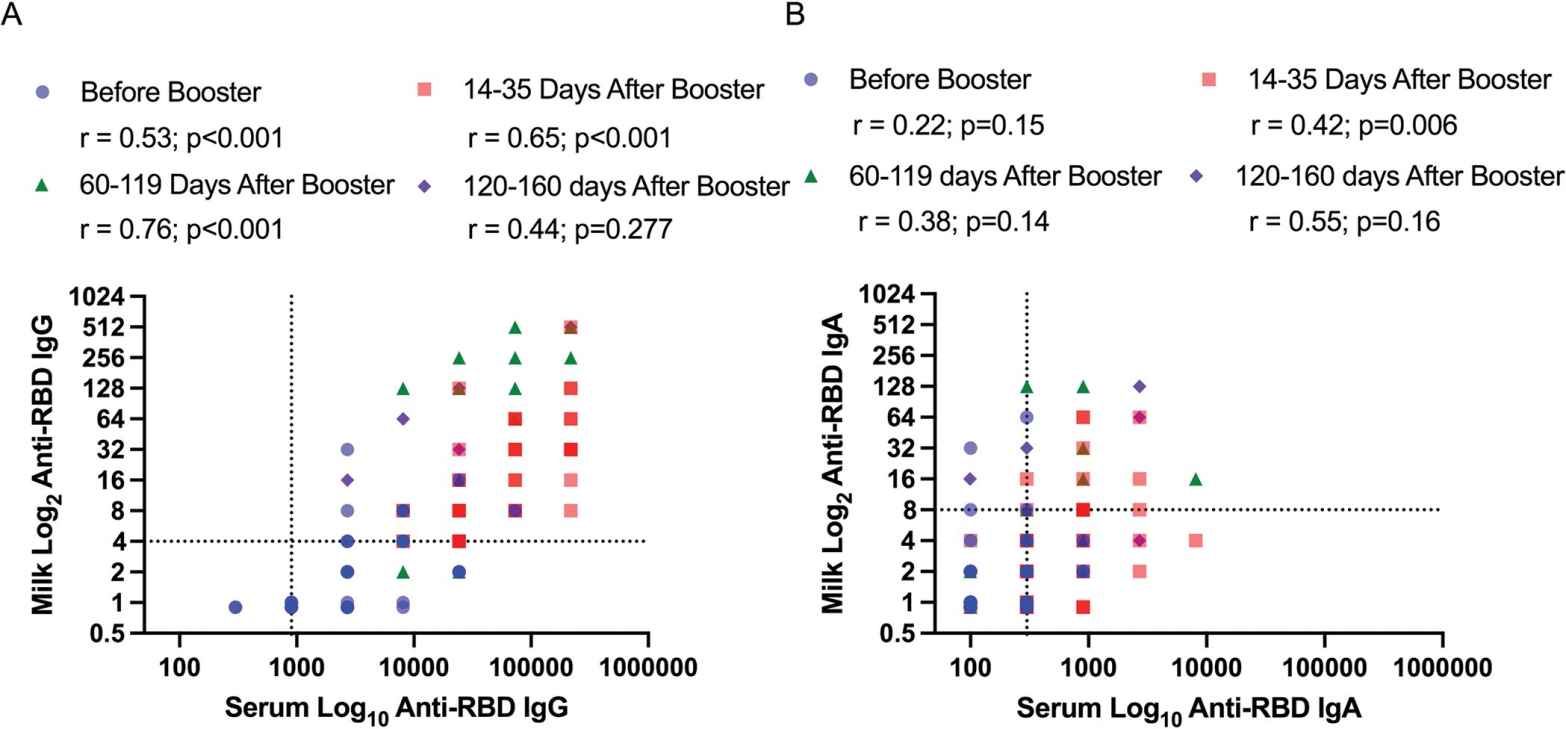
**A, B.** Correlation of human milk anti-receptor binding domain (RBD) IgG and IgA with serum IgG and IgA before and after COVID-19 booster vaccine (N = 45 paired samples). The colors represent the different time points. The dotted lines indicate the positive cutoff titer for IgG (900 for serum and 4 for milk) and IgA (300 for serum and 8 for milk). Milk IgG correlated with IgG in serum before and at 15–35 days and 60–119 days after booster vaccine (A). Milk IgA correlated with IgA in serum at 14–35 days after booster vaccine (B). Correlations were analyzed using Pearson correlation coefficient.

While milk anti-RBD IgG significantly correlated with anti-RBD IgG in paired maternal blood obtained both before and 14–35 days after booster, the correlation was strongest 60–119 days after booster (Fig 4A). Conversely, milk anti-RBD IgA only weakly correlated with maternal anti-RBD IgA in paired blood obtained 14–35 days after booster (Fig 4B).

### Anti-RBD antibodies in infant blood before and after SARS-CoV-2 booster vaccine

**Fig 5:**
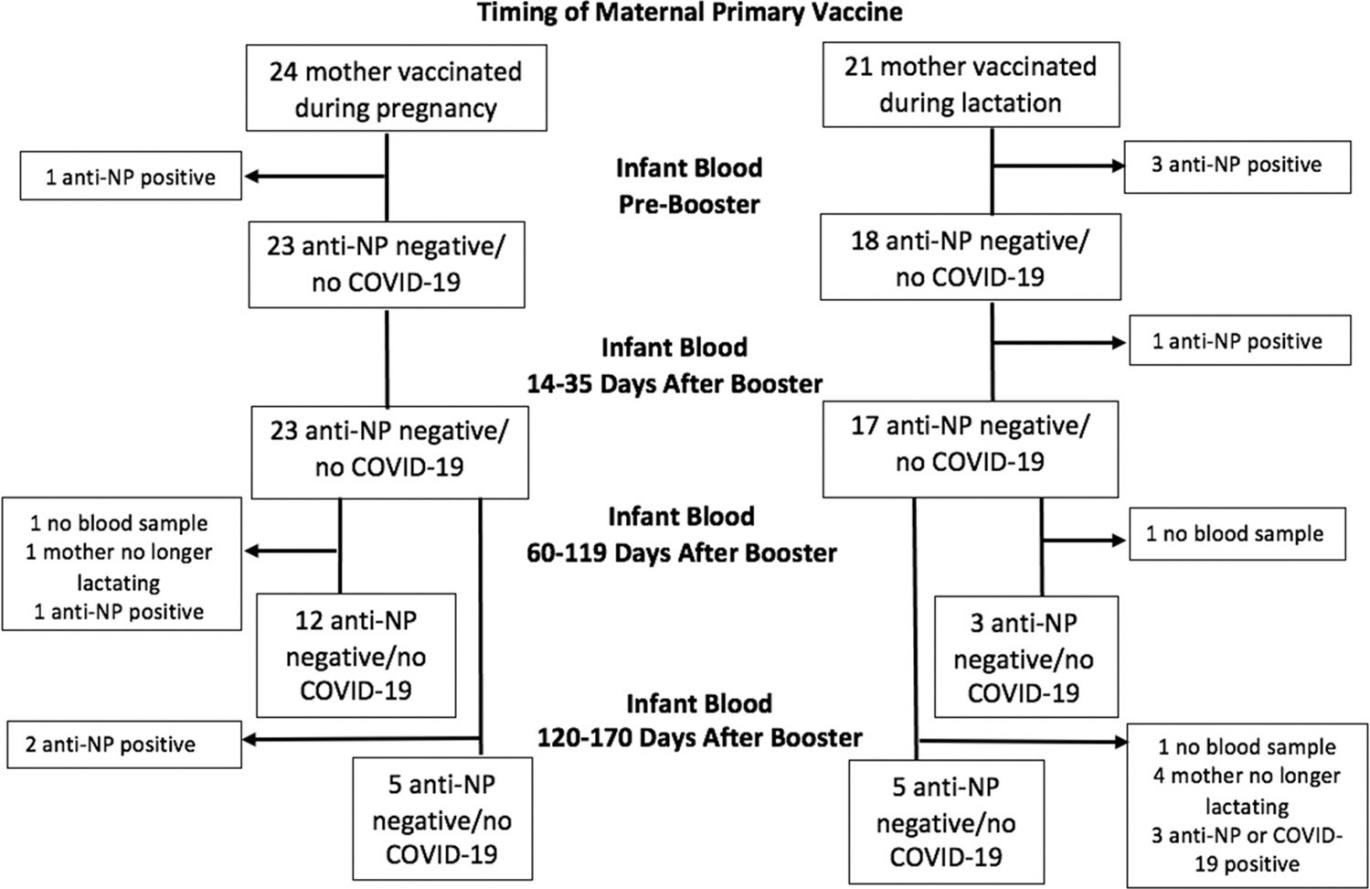
Flow chart of anti-NP results and COVID-19 infections in samples from children included in study.

Ninety-one percent (41/45) of infants were anti-NP negative on initial blood, with 9% (4/45) positive and excluded (Fig 5). Of those negative, 98% (40/41) and 81% (25/31) remained negative on their second and third blood samples, respectively.

Among anti-NP negative infants, 74% (17/23) of infants born to women vaccinated during pregnancy had positive (≥900) serum anti-RBD IgG prior to maternal booster, compared to 11% (2/18) of infants born to women vaccinated while lactating (p<0.001). Among infants born to women immunized while lactating, there was no increase in blood anti-RBD IgG after maternal booster (Fig 3E). However, among infants born to women immunized during pregnancy, there was a significant decrease in serum anti-RBD IgG at all times compared to initial blood (Fig 3E), whereby only 6% (1/17) had positive IgG by their third sample. No infants had detectable anti-RBD IgA in any blood sample (Fig 3F).

**Fig 6:**
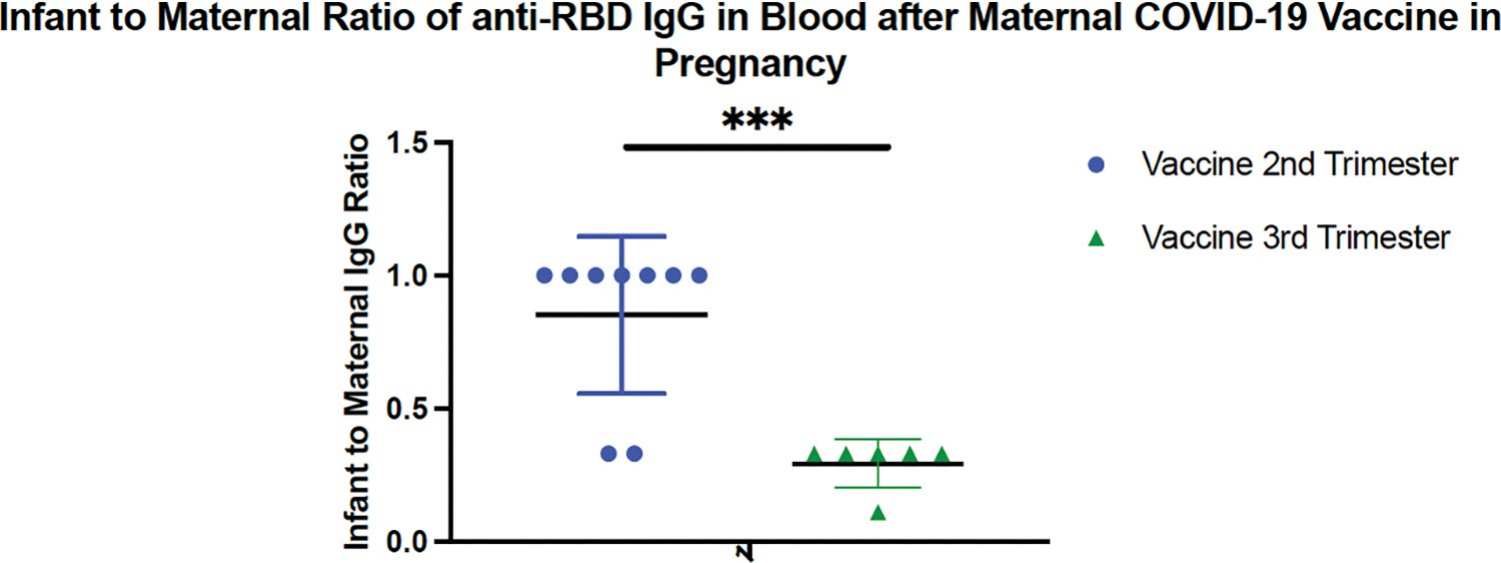
Comparison of infant to maternal anti-receptor binding domain (RBD) IgG among mother-infant pairs who are both negative for anti-NP in serum obtained before COVID-19 booster vaccine by trimester of vaccination with primary COVID-19 vaccine series (N = 15).

Among 15 mother-infant pairs who were both anti-NP negative on their first blood sample, infants born to women vaccinated during the second trimester (N = 9) had higher infant to maternal ratio of IgG GMT compared to those vaccinated during the third trimester (N = 6); mean ratio 0.85 versus 0.29; p<0.001; Fig 6). There was no difference in mean infant age at time of initial blood sample between those exposed to vaccine during the second and third trimester (5.1 versus 5.9 months, respectively; p = 0.127).

## Discussion

In this analysis, we demonstrate that lactating women who were vaccinated with SARS-CoV-2 primary series and then boosted with mRNA-based vaccine had increased breastmilk IgG and IgA compared to pre-booster milk. While this increase was detectable as early as 2 weeks after vaccination, anti-RBD titers peaked between 3–4 months and, while still elevated, began to decline by 5–6 months. There was some signal that titer levels in milk may remain higher longer for women with a history of COVID-19, but these differences were not significant, likely due to small sample size at later timepoints. While the substantial increase in milk GMT for IgA and particularly IgG after booster vaccine is encouraging, it remains unknown whether these increased titers correlate with increased protection for infants. Notably, the majority of infants born to women who received primary SARS-CoV-2 vaccine during pregnancy still had substantial transplacental antibodies five months after delivery. Our data also suggest that optimal antibody transfer may occur with second trimester vaccination. These data reinforce current recommendations for all pregnant and lactating women to receive primary and booster vaccines. Finally, we show that despite increased milk anti-RBD antibodies after maternal COVID-19 booster, there was no significant increase in anti-RBD antibodies in infant blood, indicating poor absorption of these antibodies across the gut mucosa.

Our study corroborates that maternal vaccination with mRNA vaccines may offer an important mechanism to protect infants from COVID-19 and mitigate disease severity through provision of transplacental and milk antibodies that are correlated with protection in adults. Numerous studies identified robust anti-RBD IgG and to a lesser degree IgA in milk after completion of primary COVID-19 vaccine series either during pregnancy or lactation [12, 16, 21, 22]. However, antibodies in milk wane significantly by 3–4 months after the primary series. Booster vaccines resulted in higher milk anti-RBD and neutralization antibodies up to one month after vaccination but longer term follow-up was not available [17, 23]. The increased durability of milk antibodies after booster vaccine seen in our study is likely a function of the increased durability of vaccine-induced antibodies in blood after booster. However, the etiology of the delayed peak in milk IgG after vaccination, despite similar blood titer between timepoints is less clear. While most IgG is thought to be transported into breastmilk from maternal blood by the FcRn receptor, IgG-secreting plasma cells are also present in breastmilk [24–26]. Gradual migration of plasma cells from B-cell germinal centers in surrounding lymph nodes, mammary glands, or other mucosal tissues may account for this delayed increase [26–28]. As seen with the primary series, booster vaccine induced a stronger blood and milk IgG response compared to IgA. The intramuscular route of SARS-CoV-2 vaccine administration is strongly associated with an IgG dominant antibody response [10, 12, 15, 16]. Additionally, RNA based vaccines generally favor a Th1 response compared to protein based or oral formulations, while IgA production is typically stimulated by Th2 cytokines such as IL4, IL5 and IL6 [29, 30].

The 42% seroprevalence for SARS-CoV-2 on the first maternal blood sample obtained from October 2021 to January 2022 was higher than the 20–30% prevalence previously reported in U.S. healthcare workers, although these studies were prior to the U.S. Delta wave [31–33]. While anti-NP positive women were more likely to be positive for IgG in their milk prior to booster, we did not see other differences in blood or milk GMT before or after booster by maternal NP status. The majority of these infections were likely asymptomatic or mild, which is associated with lower and shorter duration of virus-specific IgG compared to more symptomatic infections [34, 35]. Furthermore, studies on hybrid immunity following primary mRNA-based vaccines highlight that initial protection is similar between hybrid versus vaccine-only immunity. Differences in protection, including titers and neutralization, begin to emerge during long-term follow-up 6–12 months after vaccination [36, 37]. Therefore, duration of follow-up may limit our ability to identify antibody differences in blood and milk by NP status.

To evaluate whether SARS-CoV-2 antibodies in milk could be absorbed and disseminated in infant serum, we evaluated SARS-CoV-2 antibodies in infant blood following maternal booster vaccination and when milk antibodies were highest. After maternal booster, we found that no infants had increased blood anti-RBD IgG or IgA. Although transcytosis of IgG antibodies across the gastrointestinal tract from breastmilk has been described, our data does not support this as a mechanism for acquisition of serum SARS-CoV-2 antibodies in infants [38–40]. In addition to degradation of breastmilk antibodies in the gut, limiting the opportunity for transcytosis, intestinal enterocytes typically exhibit limited absorption of macromolecules in term infants [15, 26, 38, 39]. However, milk antibodies against SARS-CoV-2 may still provide meaningful protection at the mucosa by blocking virus binding, cell entry, and replication [12, 15, 17, 28, 41]. Interestingly, we identified two infants with positive serum RBD IgG GMT but negative initial anti-NP. In these cases, absorption from milk was unlikely as IgG GMT was undetectable/low on paired maternal milk samples. However, it is possible that these infants experienced SARS-CoV-2 infection but did not seroconvert for anti-NP [42, 43].

As in other studies, maternal vaccination in pregnancy is a reliable way to provide serum SARS-CoV-2 antibody to young infants and may provide critical passive immune protection until infants are eligible for COVID-19 vaccines [10, 11, 44]. Our finding of higher infant-to-maternal antibody ratios following maternal COVID-19 vaccination during the second trimester is consistent with similar studies of maternal pertussis, tetanus, and influenza vaccines [45–49].

Our study is limited by lack of respiratory surveillance with PCR testing for SARS-CoV-2 infection for participants. While we assessed for seroconversion to positive serum anti-NP antibody, the lack of surveillance data limits our ability to know if COVID-19 infection occurred or if a new infection occurred among anti-NP positive women. Additionally, our limited sample size at later timepoints and overall duration of follow-up limits some analysis of differences between women with and without hybrid immunity. Finally, the evolution of the pandemic including emergence of new variants and changes in vaccine recommendations leads to variability that could affect these findings. This includes variation in the timing of booster vaccine relative to the primary series, as many women in this study were front-line workers and among the first to receive the primary series.

## Conclusions

We demonstrate that maternal COVID-19 booster with mRNA-based vaccines generated robust and durable IgG and IgA milk antibodies. We also show that those antibodies were not detected in infant blood following booster vaccine. COVID-19 vaccination during pregnancy results in significant levels of IgG in the infant that persist up to five months and may help protect infants from COVID-19 during this vulnerable period. Further studies are needed to investigate the clinical significance of SARS-CoV-2 antibodies in milk and optimal timing of SARS-CoV-2 vaccination in pregnancy for the protection of children from COVID-19. Clinicians should use these data to encourage pregnant and lactating women to obtain primary and booster vaccines to protect themselves and their children.

## Supporting information

S1 File Detailed methods on sample processing and SARS-CoV-2 assays. (DOCX)

S2 File Deidentified dataset used for analysis. (XLSX)
