# Supplementary material for: Impact of maternal SARS-CoV-2 booster vaccination on blood and breastmilk antibodies
Source: PLoS One. 2023 Jun 13;18(6):e0287103. doi: 10.1371/journal.pone.0287103 (PMC10263312; doi:10.1371/journal.pone.0287103)
Supplement: S1 File — (DOCX) [file pone.0287103.s001.docx]

**S1. File. Detailed methods on sample processing and SARS-CoV-2 assays.**

Milk Collection

Participants collected 0.5-2 ounces of fresh milk using a hand or electric pump and transferred into a pre-labeled, sterile container. While fresh samples were preferred, frozen milk samples were allowed. Milk was then transported and frozen in a -20 Celsius (ᵒC).

Blood Collection

Blood was collected from women and their children either in the clinic setting through venipuncture or heel-stick or at-home using the TASSO-SST^©^. Clinic specimens were collected in EDTA tubes and refrigerated for up to 72 hours at 4ᵒC before processing. For home collection, up to 275 µl of whole blood was collected into a gel-lined collection tube, stored in the home refrigerator and transported to the laboratory for processing within 48 hours of collection. All blood was centrifuged for 15 minutes at room temperature at 10,000 revolutions per minute and serum was frozen in a -20ᵒC freezer.

***Anti-NP binding assays for SARS-CoV-2***. Serum samples were tested for IgG binding to SARS-COV-2 nucleoprotein (N) using indirect ELISAs. Briefly, recombinant N protein (RayBiotech) at a concentration of 2µg/ml in carbonate/bicarbonate buffer was used to coat high-binding, half-area 96- well polystyrene plates (Corning) at 4°C in a humidified chamber overnight. Plates were blocked with non-fat dry milk (NFDM) (Bio-Rad) at 5% (w/v) in 1X PBS-T for 1 hour at room temperature. Immediately after blocking, serum or plasma samples diluted in 1% (w/v) of NFDM in 1X PBS-T were added to the plate and incubated for 1 hour at room temperature. Samples were assayed in duplicate at a single dilution of 1:100. Plates were washed six times with 1X PBS-T and incubated for 1 hour with horseradish peroxidase (HRP)-linked antibody anti-human IgG (Jackson ImmunoResearch). After five washes with 1X PBS-T, plates were incubated at room temperature for 20 minutes with HRP substrate, 3, 3′, 5, 5″-tetramethylbenzidine (TMB; SeraCare) and the developing reaction was stopped by adding 1N hydrochloric acid (Sigma). Optical densities at a wavelength of 450nm (OD450nm) were measured using SpectraMax Plus PC380 microplate spectrophotometer using SoftMax Pro software version 6.4 (Molecular Devices, USA). Positive and negative control samples were included in each assay plate for cut-off values calculation. Optical densities from blank wells were subtracted from all measurements before analysis. Cut-off values were calculated as the mean OD450nm of the negative controls plus 3 times the standard deviation. The assay was optimized to detect anti-SARS-CoV-2 N IgG with sensitivity of 93.4% and specificity of 89.3% as described previously (Table S2 and Figure S2C)^1^.

***Anti-RBD binding assays for SARS-CoV-2***. MaxiSorpTM 96-well plates (Thermofisher) were coated with SARS-CoV-2 RBD-His at 50 ng per well diluted in PBS and incubated at 4 ᵒC overnight. Following removal of coating solution, plates were blocked with blocking buffer (5% nonfat milk in PBS with 0.1% Tween-20, PBST) and incubated at 37 ᵒC for 1 h. Three-fold serial dilutions of serum samples or two-fold dilutions of breastmilk samples were prepared in blocking buffer, then incubated on plates at 37 ᵒC for 2 h. Plates were washed three times with PBST followed by incubation with one of the following secondary antibodies diluted 1:10,000 in blocking buffer at 37 ᵒC for 1 h: horseradish peroxidase (HRP)-conjugated donkey anti-human IgG (Jackson ImmunoResearch), or HRP-conjugated goat anti-human IgA (Invitrogen). Plates were washed again prior to the addition of TMB peroxidase substrate mix (Seracare) and incubated at room temperature (RT) for 3-5 min. TMB stop solution (Seracare) was added and the optical density (OD) at 450 nm was measured using a Molecular Devices SpectraMax 340PC Microplate Reader. A human serum or a pre-pandemic breast milk sample was included as negative control on each plate. Samples were assayed in duplicate. Endpoint titer is defined as the dilution of sample that was 3 standard deviations above the average of the normal control samples.
